# Transition-metal (oxy)nitride photocatalysts for water splitting

**DOI:** 10.1039/d3sc03198e

**Published:** 2023-07-28

**Authors:** Kaihong Chen, Jiadong Xiao, Takashi Hisatomi, Kazunari Domen

**Affiliations:** a Research Initiative for Supra-Materials, Interdisciplinary Cluster for Cutting Edge Research, Shinshu University Nagano-shi Nagano 380-8553 Japan domen@shinshu-u.ac.jp; b PRESTO, JST 4-17-1 Wakasato, Nagano-shi Nagano 380-8553 Japan; c Office of University Professors, The University of Tokyo 2-11-16 Yayoi, Bunkyo-ku Tokyo 113-8656 Japan; d Department of Chemistry, Kyung Hee University Seoul 130-701 Republic of Korea

## Abstract

Solar-driven water splitting based on particulate semiconductor materials is studied as a technology for green hydrogen production. Transition-metal (oxy)nitride photocatalysts are promising materials for overall water splitting (OWS) *via* a one- or two-step excitation process because their band structure is suitable for water splitting under visible light. Yet, these materials suffer from low solar-to-hydrogen energy conversion efficiency (STH), mainly because of their high defect density, low charge separation and migration efficiency, sluggish surface redox reactions, and/or side reactions. Their poor thermal stability in air and under the harsh nitridation conditions required to synthesize these materials makes further material improvements difficult. Here, we review key challenges in the two different OWS systems and highlight some strategies recently identified as promising for improving photocatalytic activity. Finally, we discuss opportunities and challenges facing the future development of transition-metal (oxy)nitride-based OWS systems.

## Abstract 2

This perspective highlights recent advances and challenges in transition-metal (oxy)nitrides, which are promising photocatalysts for overall water splitting, and discusses opportunities to upgrade the solar-to-hydrogen energy conversion efficiency.

## Graphical abstract

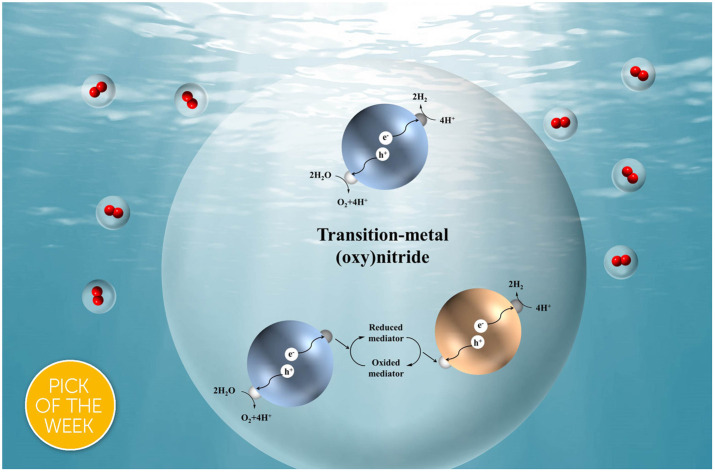

## Introduction

The conversion of solar energy into chemical fuels is an intriguing approach to alleviating energy and environmental issues. Given the high gravimetric energy density of hydrogen (H_2_), solar-driven overall water splitting (OWS) into H_2_ and oxygen (O_2_) offers a possibility to take full advantage of solar energy.^1^ To date, an apparent quantum yield (AQY) of almost unity has been achieved in OWS using an Al-doped SrTiO_3_ particulate photocatalyst facet-selectively modified with Rh/Cr_2_O_3_ and CoOOH cocatalysts *via* photodeposition.^2^ Even so, the solar-to-hydrogen energy conversion efficiency (STH) of this system is only 0.65% because Al-doped SrTiO_3_ responds solely to ultraviolet light. The STH value necessary to make this process economically competitive with H_2_ production from fossil resources has been estimated to be approximately 5–10%.^3^ Clearly, it is necessary to develop narrow-bandgap photocatalysts that have a longer absorption-edge wavelength to effective utilize visible components in sunlight.^4,5^

Compared with metal oxide semiconductors, (oxy)nitride semiconductors have narrower bandgaps because N 2p orbitals have a more negative potential energy than O 2p orbitals and because the valence-band edge is shifted negatively.^6^ As a result, the absorption edges of these (oxy)nitride materials are mostly extended to the visible-light range while band-edge potentials suitable for OWS are maintained.^7–10^ For instance, with increasing N content, the absorption-edge wavelengths become longer: from ∼320 nm for Ta_2_O_5_ to ∼500 nm for TaON and ∼600 nm for Ta_3_N_5_. Thermodynamically, all of these materials can evolve both H_2_ and O_2_ from an aqueous solution.^11^ Note that nitrogen-doped transition-metal oxides have been reviewed elsewhere^12,13^ and will not be discussed in this perspective, because N atoms only form impurity (discontinuous) levels in these materials.

Apart from the extension of the absorption edge, charge separation and migration should also be improved and the desired surface redox reactions should be enhanced to increase the OWS activity.^11^ However, the situation is complicated and often difficult to control when transition-metal (oxy)nitride-based photocatalysts are used. First, anion vacancies and reduced species (*e.g.*, Ta^4+/3+^ or Ti^3+^) are inevitably formed during high-temperature nitridation under flowing ammonia (NH_3_) because of the decomposition of NH_3_ into N_2_ and H_2_ at high temperatures. Such defects can function as recombination centers, decreasing the number of surviving photoexcited charge carriers and reducing the photocatalytic activity. In addition, a complicated nitridation process (anion exchange and rearrangement) also limits the availability of (oxy)nitrides with well-defined facets. Moreover, the poor thermal stability of (oxy)nitrides at elevated temperatures in air is a large obstacle to the design of effective cocatalyst loading procedures. Even though most oxynitride materials are expensive, the material cost of the photocatalyst is estimated to be insignificant compared with the cost of the photocatalytic reactor due to the small amount of the photocatalyst loaded, and therefore will be allowable.^3^

Numerous transition-metal (oxy)nitrides have been shown to exhibit activities toward both the H_2_ evolution reaction (HER) and the O_2_ evolution reaction (OER) in the presence of sacrificial electron donors and acceptors, respectively.^7^ However, very few of them have shown activity in the one-step-excitation or two-step-excitation (*i.e.*, Z-scheme process) OWS reaction. In addition, in the few known cases of OWS-active transition-metal (oxy)nitride photocatalysts, the AQY is relatively low. For example, a BaTaO_2_N-based photocatalyst with a bandgap of 1.9 eV was recently reported to achieve OWS *via* one-step excitation; however, the AQY was less than 0.1% at 420 nm.^14^ In fact, the STH value for an OWS process driven solely by (oxy)nitrides has not exceeded 0.3% yet, irrespective of whether one-step or two-step excitation systems are used. This poor photocatalytic performance is partially attributable to the surface reduction or oxidation reactions being much more sluggish in the absence of sacrificial reagents, which allows unreacted electrons and holes to simply recombine.

The development of strategies that can increase the STH value for OWS using (oxy)nitride photocatalysts is critical and urgently needed. In this perspective, we summarize some key strategies developed for enhancing OWS activity by one- or two-step excitation schemes using transition-metal (oxy)nitride photocatalysts. Our goal is to provide a springboard for further research.

## Two OWS systems

**Fig. 1:**
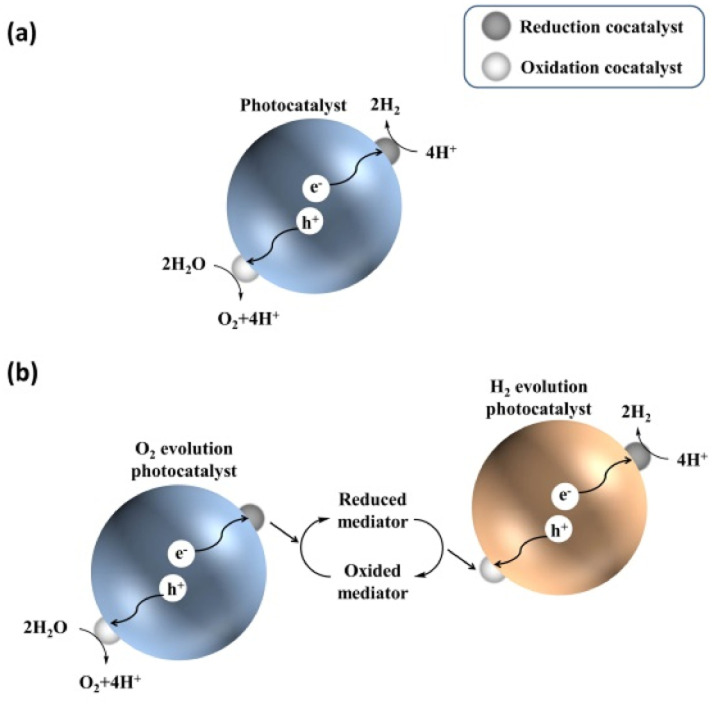
Schematics of (a) one-step-excitation and (b) two-step-excitation (Z-scheme) overall water-splitting processes.

Before describing challenges and strategies for enhancing the OWS activity of photocatalytic systems based on transition-metal (oxy)nitrides, basic aspects of one- and two-step-excitation OWS systems are introduced because the reaction properties required for the respective systems differ. The one-step-excitation OWS reaction has been studied intensively for decades as a simple and readily scaled-up approach.^15–18^ In this scheme, the bandgap of the semiconductor photocatalyst should straddle both the HER and OER potentials (Fig. 1a). In addition, because the HER and OER occur on the same photocatalyst, the photocatalysts need to be carefully designed to promote spatial charge separation and HER/OER processes while suppressing the water-formation reaction. To this end, in most cases, several strategies are applied concurrently to realize the one-step-excitation OWS reaction.

Inspired by natural photosynthesis, a typical Z-scheme OWS system comprises a H_2_-evolution photocatalyst (HEP), an O_2_-evolution photocatalyst (OEP), and shuttle redox mediators.^19,20^ As shown in Fig. 1b, the HEP and OEP produce H_2_ and O_2_ separately and the redox mediator functions as an electron mediator between the HEP and OEP. Photocatalysts can be used in Z-scheme OWS as long as they are active either toward the HER or the OER; therefore, the bandgap of the photocatalyst is not required to straddle both the HER and OER potentials. This approach provides opportunities for narrow-bandgap photocatalysts to participate in OWS under visible light. However, this feature utilizing a cascade of electron flow also provides opportunities for backward electron transfer. Undesirable backward reactions involving redox couples, in addition to those involving the water-formation reaction, should therefore be suppressed.

## Z-scheme OWS systems

We first describe progress and challenges associated with Z-scheme OWS despite the complexity of the system, because many strategies originally developed to promote half-reactions have been effectively exploited for Z-scheme OWS prior to the realization of one-step-excitation water splitting. In this section, we highlight some representative strategies such as reducing defect densities, promoting charge separation and migration, and loading cocatalysts to promote surface redox reactions.

### Reduction of defect densities

**Fig. 2:**
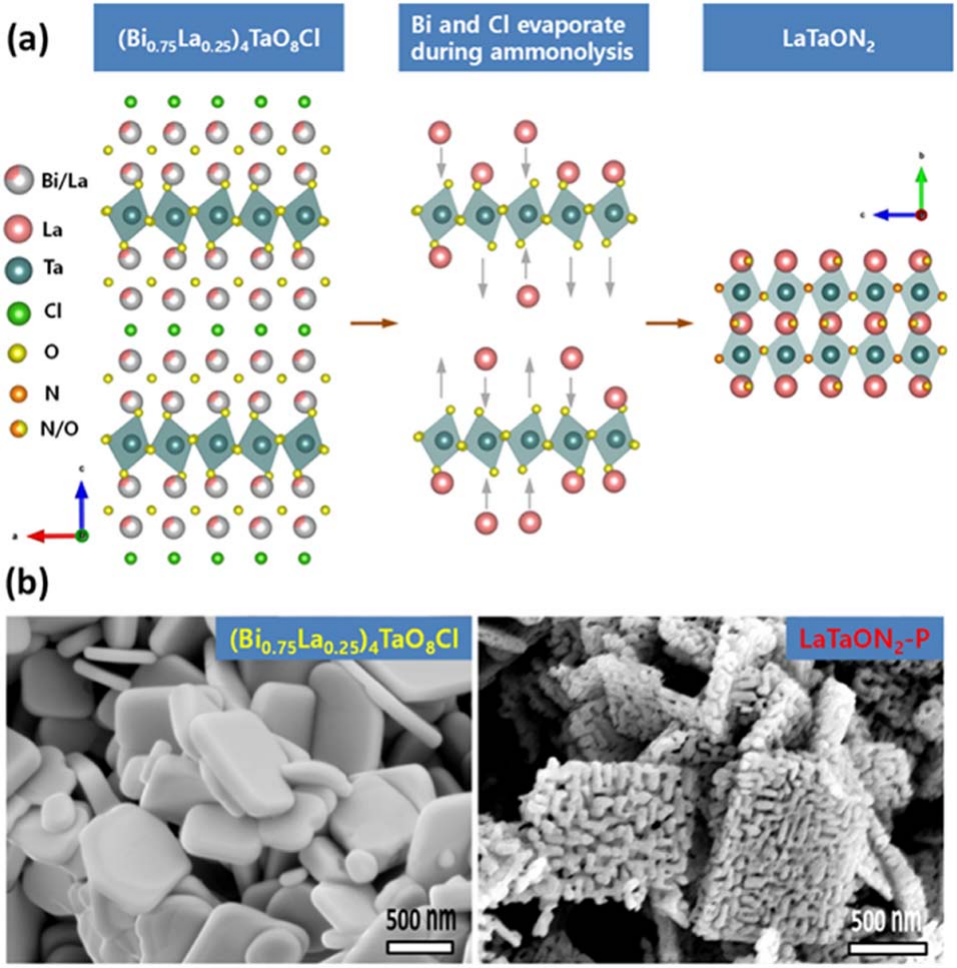
(a) Schematic of structural transformation from (Bi_0.75_La_0.25_)_4_TaO_8_Cl into LaTaO_2_N. (b) Field-emission scanning electron microscopy images of (Bi_0.75_La_0.25_)_4_TaO_8_Cl and LaTaON_2_-P. Adapted with permission from ref. 21. Copyright 2021 American Chemical Society.

The synthesis of (oxy)nitrides by NH_3_ nitridation at high temperature tends to generate defects both in the bulk and at the surface. Decreasing the defect density is an important target for transition-metal (oxy)nitrides used in OWS. One reason for the formation of defects is the structure difference between the precursors and the target products. In this context, a radical redesign of the starting materials and synthesis procedure could be beneficial. Xu *et al.* designed some double-layer Sillén–Aurivillius-type compounds, in which the Aurivillius units contain perovskite-type blocks topotactically similar to (oxy)nitrides (Fig. 2a), as precursors.^21–23^ (Bi_0.75_La_0.25_)_4_TaO_8_Cl can be transformed into LaTaO_2_N (denoted as LaTaO_2_N-P) under high-temperature nitridation.^21^ During the nitridation, Bi^3+^ and Cl^−^ ions evaporate and the residual perovskite-type blocks are converted to LaTaO_2_N without a drastic rearrangement of the atoms. Meanwhile, the evaporation of Bi^3+^ and Cl^−^ ions can form voids in the particles to facilitate replacement of O by N, which helps suppress the reduction of Ta^5+^ (Fig. 2b).^21^ After CoO_*x*_ was loaded as an O_2_-evolution cocatalyst (OEC), the OER activity of such porous LaTaO_2_N was six times greater than that of LaTaO_2_N prepared from LaTaO_4_. When combined with a Ru/SrTiO_3_:Rh HEP and a Fe^3+^/Fe^2+^ redox mediator, the porous LaTaO_2_N could split water under visible light *via* the Z-scheme. Moreover, this topotactic conversion strategy has been extended to the synthesis of SrTaO_2_N and SrNbO_2_N.^22,23^

The addition of certain additives to precursors can also afford the desired transition-metal (oxy)nitride photocatalysts. Recently, the flux-assisted nitridation technique has emerged as a powerful method to prepare (oxy)nitrides with controllable crystallinity, morphology, surface features, and particle size. Li *et al.* discovered that BaTaO_2_N prepared *via* a flux method exhibits weaker background absorption in the 700–800 nm range, indicating a decreased defect density compared with BaTaO_2_N nitrided in the absence of a flux.^24^ Using a LiBa_4_Ta_3_O_12_ oxysalt as a precursor, in which the Li^+^ was easily evaporated, they fabricated BaTaO_2_N (denoted as BaTaO_2_N-flux) with a porous structure and low defect density.^24^ A Z-scheme OWS reaction using the Pt/BaTaO_2_N-flux as the HEP, PtO_*x*_/WO_3_ as the OEP, and IO_3_^−^/I^−^ as the redox mediator exhibited twofold greater activity than an analogous system based on BaTaO_2_N synthesized without the flux.

Another example reported by Zhang and coworkers is the addition of Mg powder during the nitridation of YTaO_4−*x*_N_*y*_ from YTaO_4_.^25^ The added Mg increased the N content in the nitridation product and extended the absorption edge toward longer wavelengths. This effect was attributed to Mg functioning as a reducing reagent to weaken the metal–oxygen bond in YTaO_4_ to facilitate the replacement of O atoms by N atoms. Using Pt/YTaO_4−*x*_N_*y*_ as the HEP, PtO_*x*_/WO_3_ as the OEP, and IO_3_^−^/I^−^ as the redox mediator, the authors constructed a Z-scheme system that could split water into H_2_ and O_2_ under visible light.

The second approach is constructing solid solutions between two different semiconductors to tune the bandgap energy. In 2011, Maeda *et al.* prepared a BaZrO_3_–BaTaO_2_N solid solution by nitriding a mixture of BaZrO_*x*_ and BaTaO_*x*_.^26,27^ Because of the incorporation of the BaZrO_3_ component, this solid solution had a larger bandgap than pure BaTaO_2_N and demonstrated a stronger driving force for the HER and OER. In addition, the defect density in the BaZrO_3_–BaTaO_2_N solid solution decreased because the background absorption beyond the absorption-edge wavelength (650 nm) decreased. After being modified with Pt as a H_2_-evolution cocatalyst (HEC), the BaZrO_3_–BaTaO_2_N solid solution exhibited enhanced HER activity. It was applicable to Z-scheme OWS; however, the AQY at 420–440 nm was less than 0.1%. Similarly, SrZrO_3_ was found to enlarge the bandgap and reduce the defect density of LaTaON_2_ by forming solid solutions.^28^ Compared with pristine LaTaON_2_, the LaTaON_2_–SrZrO_3_ solid solution exhibited enhanced OER activity. Moreover, when coupled with Ru/SrTiO_3_:Rh in FeCl_3_ solution, the LaTaON_2_–SrZrO_3_ solid solution split water into stoichiometric H_2_ and O_2_ under visible light *via* a Z-scheme process.

Doping is also a prevalent strategy in the preparation of photocatalysts. The introduction of foreign elements enables control of the optical, particle, and semiconducting properties. For example, doping BaTaO_2_N with a moderate amount of Zr was shown to enhance the H_2_ evolution of Na–Pt/BaTaO_2_N.^29^ Analysis by transient absorption spectroscopy (TAS) showed that doping with 1 mol% Zr could increase the population of shallowly trapped electrons and that increasing the amount of Zr to 10 mol% reduced both the electron and hole densities. When combined with CoO_*x*_/Au/BiVO_4_ as the OEP and [Fe(CN)_6_]^3−^/[Fe(CN)_6_]^4−^ as the redox mediator, Na–Pt/BaTaO_2_N:Zr exhibited an AQY of 1.5% at 420 nm and an STH of 2.2 × 10^−2^% in Z-scheme OWS.

### Promotion of charge separation and migration

The efficiency of photocatalysis is affected by both carrier recombination and charge separation/migration. The anisotropic nature of some semiconductors, such as SrTiO_3_ and BiVO_4_, can provide materials with a sufficient driving force for the separation and transportation of photogenerated electrons and holes toward surface reduction and oxidation sites, respectively.^30,31^ The facet-selective loading of cocatalysts onto these materials can substantially reduce the charge recombination probability and improve the charge separation efficiency. As previously mentioned, the difficulty in constructing transition-metal (oxy)nitrides with high crystallinity and anisotropic facet exposure limits the applicability of this strategy. Nevertheless, recent studies have suggested that flux-assisted nitridation can overcome this limitation. Luo *et al.* showed that {100} and {110} facets can be exposed simultaneously on BaTaO_2_N when a KCl flux is used.^32^ In addition, Pt nanoparticles can be selectively photo-deposited onto the {100} facets. This feature enhanced the charge separation of BaTaO_2_N, leading to tenfold greater HER activity compared with that of BaTaO_2_N with only its {100} facets exposed.^32^ This observation suggests that facet-selective cocatalyst loading methods can be applied to transition-metal (oxy)nitrides.

**Fig. 3:**
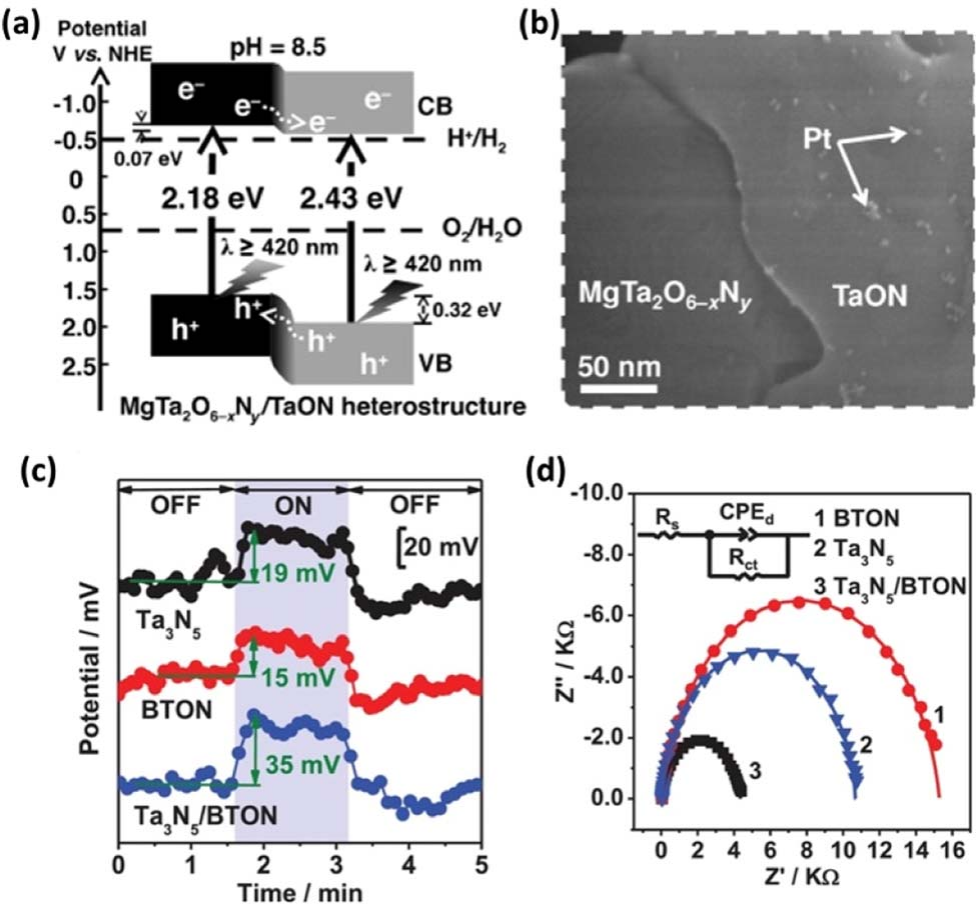
(a) Estimated band positions for the MgTa_2_O_6−*x*_N_*y*_/TaON heterostructure. (b) Field-emission scanning electron microscopy images of the Pt–MgTa_2_O_6−*x*_N_*y*_/TaON photocatalyst. Adapted with permission from ref. 33. Copyright 2015 Wiley-VCH. (c) Surface potentials of three typical BTON in the dark and under irradiation with 450 nm light. (d) EIS results for three BTON measured at 1.23 V *vs.* the reversible hydrogen electrode (RHE). Adapted with permission from ref. 34. Copyright 2019 Wiley-VCH.

A heterostructure system can promote photoexcited electron and hole transfer in different directions. Such systems are also often effective for forming heterojunctions promoting the functionality of transition-metal (oxy)nitride photocatalysts. Chen *et al.* prepared a MgTa_2_O_6−*x*_N_*y*_/TaON heterostructure *via* the one-pot nitridation of MgTa_2_O_6_/Ta_2_O_5_ (Fig. 3a).^33^ Because the photodeposition of Pt nanoparticles mainly occurred on the TaON surface (Fig. 3b), the authors suggested that photogenerated electrons migrated toward the conduction-band minimum of TaON while holes migrated to the valence-band maximum of MgTa_2_O_6−*x*_N_*y*_. Benefitting from the effective interfacial charge separation and reduced defect density, the AQY for Z-scheme OWS involving MgTa_2_O_6−*x*_N_*y*_/TaON reached 6.8% at 420 nm. This strategy has also been extended to similar bi-(oxy)nitride heterojunctions such as BaTaO_2_N/Ta_3_N_5_,^34,35^ BaMg_1/3_Ta_2/3_O_3−*x*_N_*y*_/Ta_3_N_5_,^36^ and CaTaO_2_N/Ta_3_N_5_.^37^ Analyses using Kelvin probe force microscopy (KPFM) and electrochemical impedance spectroscopy (EIS) demonstrated enhanced charge separation in the BaTaO_2_N/Ta_3_N_5_ and CaTaO_2_N/Ta_3_N_5_ heterojunction systems (Fig. 3c and d) according to the authors of the articles.^34,37^

### Cocatalyst design promoting surface redox reactions

The cocatalyst is also an important component of OWS systems. Cocatalysts are expected to promote not only charge separation and transfer but also surface redox reactions. This function will, in turn, necessitate intimate interfacial contact between semiconductors and cocatalysts *via* a suitable junction. However, (oxy)nitrides might decompose into oxides at high temperatures in air. To avoid the decomposition of (oxy)nitrides during the loading of cocatalysts, Zhang *et al.* devised a method to load CoO_*x*_ as an OEC onto (oxy)nitrides under a high-temperature NH_3_ flow.^38^ Strong interfacial interaction occurred between the Co species and LaTiO_2_N, without destruction of the oxynitride. The resultant CoO_*x*_/LaTiO_2_N exhibited an excellent AQY in the OER (27.1 ± 2.6% at 440 nm).

**Fig. 4:**
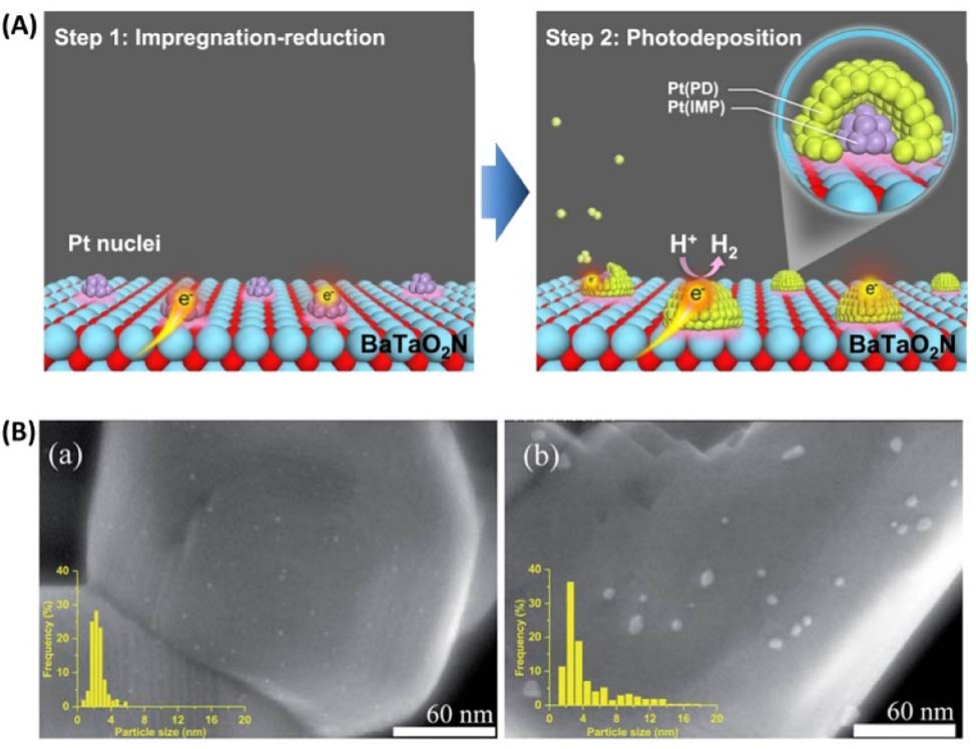
(A) Schematic of sequential Pt cocatalyst deposition onto BaTaO_2_N. Adapted with permission from ref. 40. Copyright 2021 Springer Nature. (B) Scanning transmission electron microscopy images and particle size distributions of (a) Na-containing and (b) Na-free Pt/BaTaO_2_N. Adapted with permission from ref. 41. Copyright 2021 The Royal Society of Chemistry.

Concerning the HER, a sequential cocatalyst loading procedure (*i.e.*, impregnation–reduction, followed by photodeposition) was recently developed to modify Ta_3_N_5_ and BaTaO_2_N with highly dispersed and intimately contacted Pt nanoparticles (Fig. 4A).^39,40^ The HER activity of the resultant Pt/BaTaO_2_N was enhanced almost threefold compared with the case of BaTaO_2_N modified with Pt *via* an impregnation–reduction procedure.^40^ Z-scheme OWS using Pt/BaTaO_2_N as the HEP and PtO_*x*_/WO_3_ as the OEP exhibited an AQY of 4.0% at 420 nm and an STH of 0.24%.^40^ Moreover, electron transfer from BaTaO_2_N to the Pt nanoparticles was verified *via* TAS experiments. The absorption intensity at 5000 cm^−1^ (2000 nm, 0.62 eV) for Pt/BaTaO_2_N decayed faster when Pt was loaded *via* a two-step procedure than when it was loaded *via* only a single-step impregnation–reduction procedure or *via* photodeposition. Notably, the HER activity of Pt/BaTaO_2_N was well correlated with the OWS activity of the Z-scheme systems, indicating that further improvements in the performance of the HEP based on Pt/BaTaO_2_N will improve the OWS activity of this Z-scheme system.^40^ Adding a small amount of Na^+^ during the impregnation of Pt is another method to fabricate well-dispersed Pt nanoparticles on the surface of BaTaO_2_N (Fig. 4B), resulting in Pt/BaTaO_2_N with improved HER activity.^41^

**Fig. 5:**
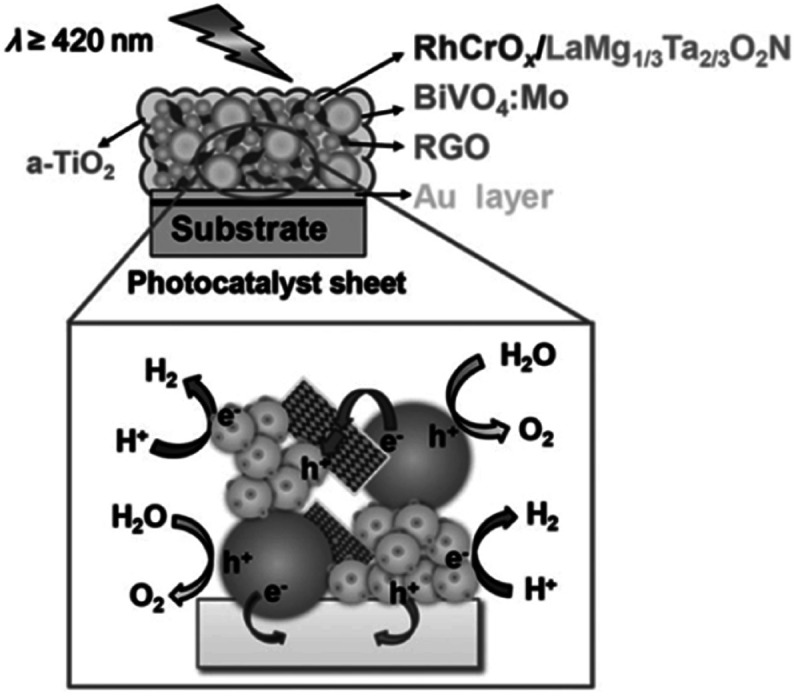
Schematic of a-TiO_2_-coated (RhCrO_*x*_/ZrO_2_/LaMg_1/3_Ta_2/3_O_2_N)/(Au, RGO)/BiVO_4_:Mo photocatalyst sheet. Adapted with permission from ref. 44. Copyright 2016 Wiley-VCH.

In an ideal Z-scheme system, ionic redox mediators can transfer electrons and holes between two different photocatalysts. However, backward reactions involving redox mediators generally suppress the OWS activity because these side reactions are thermodynamically more favorable. Moreover, the activity of Z-scheme OWS systems is, in most cases, highly sensitive to the kinds and concentrations of the redox mediators. As an example, IrO_2_/TaON can oxidize water to O_2_ in the presence of AgNO_3_ as a sacrificial electron acceptor; however, it exhibits almost no OER activity in an aqueous NaIO_3_ solution, likely because of preferential oxidation of the generated I^−^ ions.^42^ To avoid problems arising from reversible redox mediators, solid electron conductors such as reduced graphene oxide (RGO) and Au have been applied to Z-scheme systems involving transition-metal (oxy)nitrides. Z-scheme photocatalyst sheets consisting of RhCrO_*x*_/ZrO_2_/LaMg_1/3_Ta_2/3_O_2_N as the HEP and BiVO_4_:Mo as the OEP embedded in a Au layer have been reported to split water under visible-light irradiation; however, the STH is still low (∼1 × 10^−3^%).^43^ The STH was slightly improved to 3.5 × 10^−3^% when RGO was introduced as an additional solid electron conductor into this system; this improvement was attributed to an enhancement in the efficiency of charge transfer between the photocatalytic particles (Fig. 5).^44^ Moreover, (oxy)nitrides can be used as the OEP in a Z-scheme sheet system.^45^ A photocatalyst sheet based on Ga-doped La_5_Ti_2_Cu_0.9_Ag_0.1_O_7_S_5_ as the HEP and CoO_*x*_/LaTiO_2_N as the OEP embedded in a thin Au film split water with an AQY in the order of 10^−2^% at 420 nm. In this case, CoO_*x*_ was suggested to promote not only the OER on LaTiO_2_N but also electron transfer from LaTiO_2_N to Au.^45^

## One-step-excitation OWS systems

Compared with the Z-scheme OWS system, the one-step-excitation OWS reaction is a relatively simpler system that uses only one semiconductor material as a photocatalyst. Nevertheless, because of the rigorous requirements for the one-step-excitation OWS procedure, only a limited number of (oxy)nitrides have achieved OWS *via* such a mode. Herein, we first highlight a typical example that combines band engineering and surface modification to achieve one-step-excitation OWS by using an (oxy)nitride photocatalyst for the first time. We then focus on cases based on a dual-cocatalyst strategy that have recently been widely applied.

### Combination of band engineering and surface modification

**Fig. 6:**
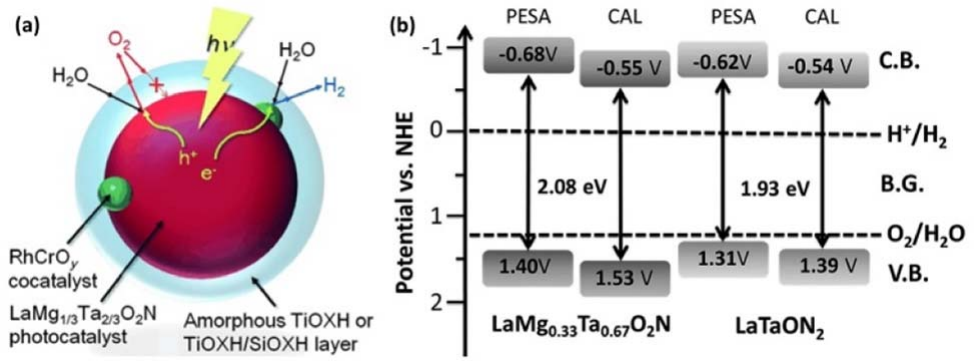
(a) Schematic of the OWS reaction mechanism on surface-coated RhCrO_*x*_/LaMg_1/3_Ta_2/3_O_2_N. Adapted with permission from ref. 46. Copyright 2015 Wiley-VCH. (b) Band levels for LaMg_*x*_Nb_1−*x*_O_1+3*x*_N_2−3*x*_ (*x* = 0 and 0.33) estimated by theoretical calculations (CAL) and photoelectron spectroscopy in air (PESA). Adapted with permission from ref. 47. Copyright 2016 The Royal Society of Chemistry.

In 2015, our group reported, for the first time, one-step-excitation OWS under visible light with wavelengths as long as 600 nm using an LaMg_1/3_Ta_2/3_O_2_N photocatalyst (Fig. 6a).^46^ In this case, both band engineering and surface modification were used. Through the formation of solid solutions of two perovskite-type semiconductors (LaTaON_2_ and LaMg_2/3_Ta_1/3_O_3_), the absorption-edge wavelength of LaMg_*x*_Ta_1−*x*_O_1+3*x*_N_2−3*x*_ varied from 525 to 640 nm with a decreasing value of *x* from 0.6 to 0 because of the increase in the O-to-N ratio in conjunction with the substitution of Mg^2+^ for Ta^5+^. According to theoretical calculations and a photoelectron spectroscopy investigation, the valence-band maximum was shifted toward more positive potentials, leading to an improvement in both the OER and OWS reaction (Fig. 6b).^47^ Mg^2+^ was also found to tune the bandgap of LaMg_*x*_Nb_1−*x*_O_1+3*x*_N_2−3*x*_; the OWS reaction could only be achieved when a suitable amount of Mg^2+^ was present.^48^

Recently, the substitution of Ca^2+^ for La^3+^ in LaTaO_2_N was investigated to modulate the band structure and defect concentration *via* the formation of a (LaTaON_2_)_1−*x*_(CaTaO_2_N)_*x*_ solid solution.^49^ After the optimized La_0.1_Ca_0.9_TaO_1+*y*_N_2−*y*_ was decorated with RhCrO_*x*_ as a cocatalyst, it exhibited OWS activity, with an AQY of ∼0.06% at ∼420 nm. In contrast to La_0.1_Ca_0.9_TaO_1+*y*_N_2-*y*_, neither LaTaO_2_N nor CaTaO_2_N showed activity toward the OWS reaction when used as a photocatalyst. Our group reported one-step-excitation OWS using CaTaO_2_N modified with RhCrO_*x*_; however, the AQY was low (on the order of 10^−3^% at 440 nm).^50^

The surface of LaMg_1/3_Ta_2/3_O_2_N was also modified with a nanolayer of amorphous oxyhydroxide (OXH).^46,51^ Photodegradation of the (oxy)nitride and the water-formation reaction were both suppressed after the entire RhCrO_*x*_/LaMg_1/3_Ta_2/3_O_2_N surface was coated with a TiOXH layer. Such a deposited OXH nanolayer can function as a molecular sieve, where H_2_ and O_2_ evolved on the surface of RhCrO_*x*_/LaMg_1/3_Ta_2/3_O_2_N can migrate to the outer phase, whereas migration in the opposite direction is inhibited. As a result, backward reactions involving the O_2_ reduction reaction are effectively suppressed. Moreover, compared with RhCrO_*x*_/LaMg_1/3_Ta_2/3_O_2_N coated only with a TiOXH layer, RhCrO_*x*_/LaMg_1/3_Ta_2/3_O_2_N coated with both TiOXH and SiOXH layers showed enhanced OWS activity.^51^ We speculated that the double coating layer was more uniform because SiOXH could increase the hydrophilicity. The AQY at 440 ± 30 nm was increased from ∼3 × 10^−2^% to 0.18% by optimization of the nanolayer precursor and deposition procedure.

### Strategies based on dual cocatalysts

**Fig. 7:**
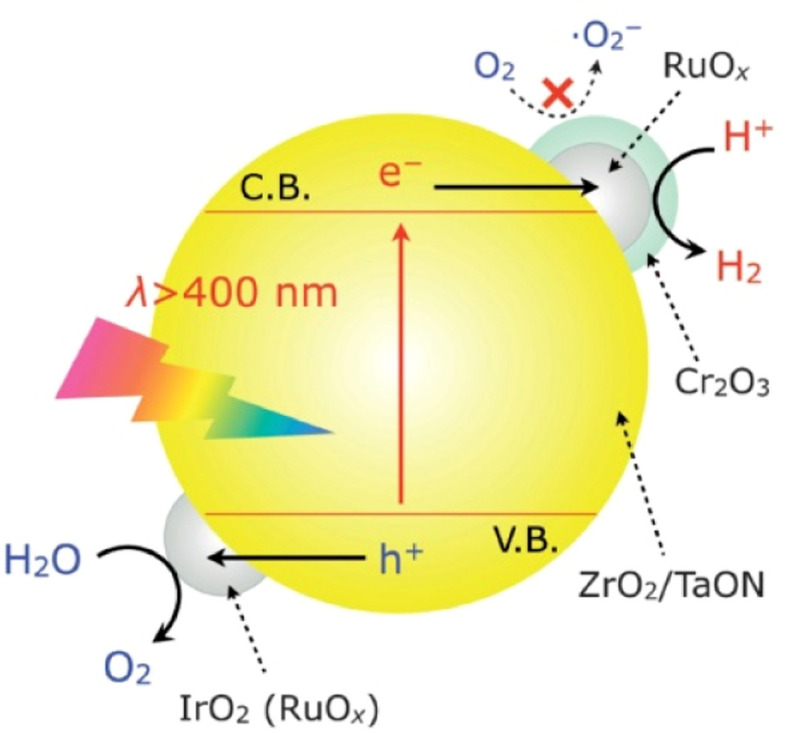
Schematic of the OWS mechanism on IrO_2_/Cr_2_O_3_/RuO_*x*_/ZrO_2_/TaON. Adapted with permission from ref. 55. Copyright 2013 Wiley-VCH.

A dual-cocatalyst strategy is another prevalent method used to realize one-step-excitation OWS reactions.^52–54^ Because the surface HER and OER are sluggish, enhancing both the HER and OER simultaneously is critical, particularly for narrow-bandgap oxynitrides. In this context, the adsorption of colloidal IrO_2_ onto the surface of an oxynitride semiconductor surface is a feasible approach. For example, IrO_2_/Cr_2_O_3_/RuO_*x*_/ZrO_2_/TaON has been reported to split water into H_2_ and O_2_ in a stoichiometric ratio under visible-light irradiation; however, the AQY was less than 0.1% at 420 nm (Fig. 7).^55^ In this system, Cr_2_O_3_/RuO_*x*_ can extract photogenerated electrons from ZrO_2_/TaON while IrO_2_ provides water oxidation sites that consume photogenerated holes. For comparison, ZrO_2_/TaON loaded with only the HEC (*i.e.*, Cr_2_O_3_/RuO_*x*_/ZrO_2_/TaON) exhibited relatively lower activity and stability for OWS. Notably, TaON without the ZrO_2_ modification could not split water; modifying the surface of TaON with ZrO_2_ nanoparticles to suppress the formation of surface defects (Ta^3+^ or Ta^4+^) was indispensable for realizing OWS. Recently, Zr-doped TaON (TaON:Zr) was fabricated using small, amorphous Ta_2_O_5_·3.3H_2_O particles as a precursor.^56^ As a result of a substantially reduced particle size by precursor design and a lowered defect density by Zr doping, IrO_2_/Cr_2_O_3_/Ru/TaON:Zr achieved greater AQY (420 nm) and STH values of 0.66% and 9 × 10^−3^%, respectively. Similarly, IrO_2_/Cr_2_O_3_/Rh/BaTaO_2_N:Mg, which features combined dual cocatalyst loading and lower-valent-cation doping, achieved one-step-excitation OWS with an AQY of 0.08% at 420 nm and an STH of 4 × 10^−4^%.^14^ Notably, BaTaO_2_N:Mg has the smallest bandgap (∼1.9 eV) among the materials that have achieved one-step-excitation OWS.

**Fig. 8:**
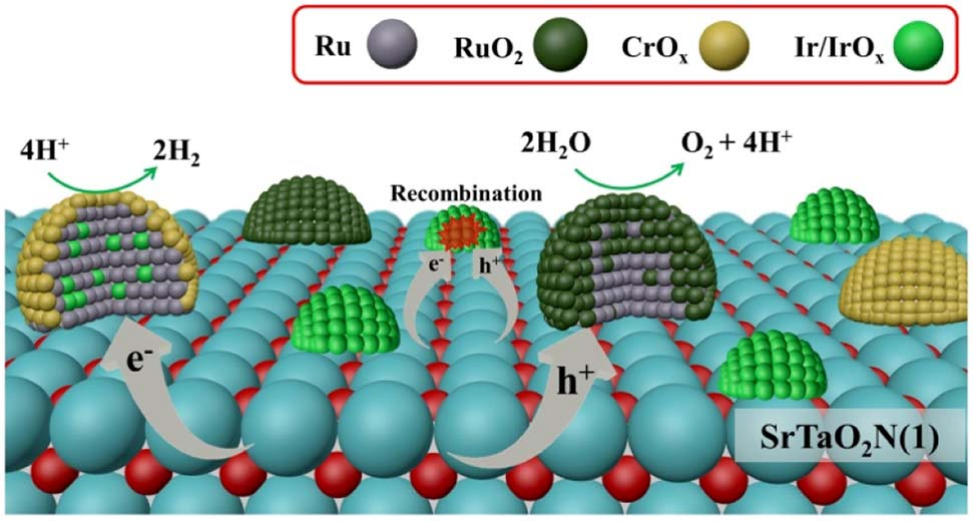
Schematic of dispersion of cocatalysts and dominant charge transfer processes on the SrTaO_2_N surface. Adapted with permission from ref. 57. Copyright 2023 American Chemical Society.

In addition to the use of colloidal IrO_2_, other strategies have been developed to fabricate dual cocatalysts. Recently, bimetallic nanoparticle cocatalysts were loaded onto a SrTaO_2_N-based photocatalyst using microwave-assisted heating (Fig. 8).^57^ Specifically, highly dispersed IrO_2_ nanoparticles were deposited through microwave-assisted heating. Subsequent loading of Ru species by impregnation and H_2_ reduction produced bimetallic RuIrO_*x*_ nanoparticles. The resultant bimetallic nanoparticles were found to effectively extract electrons from semiconductors and accelerate the HER. Coexisting RuO_*x*_ served as an OEC. The modified SrTaO_2_N-based photocatalyst exhibited an STH value of 6.3 × 10^−3^% and an AQY (420 ± 30 nm) of 0.34% in the OWS reaction.^57^

**Fig. 9:**
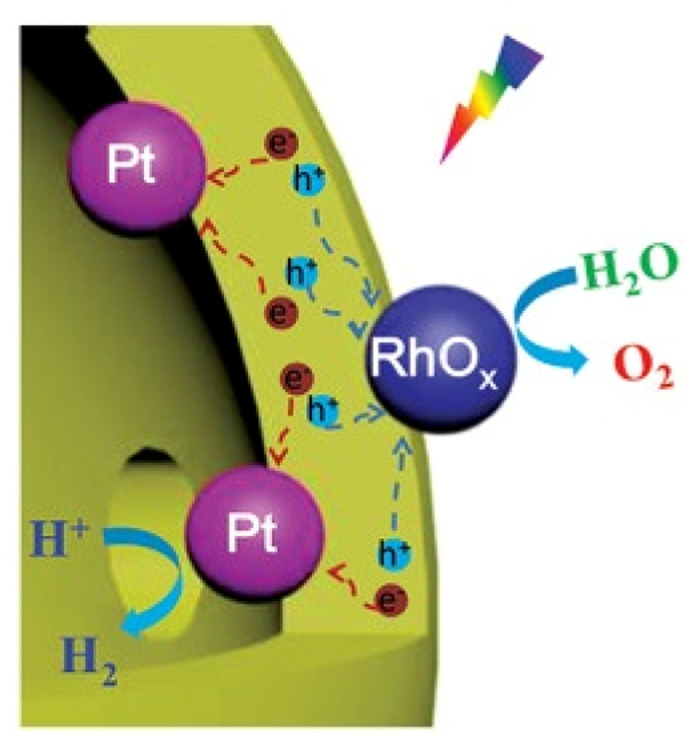
Schematic of charge separation processes on Pt@ZnTiO_3−*x*_N_*y*_@RhO_*x*_. Adapted with permission from ref. 59. Copyright 2021 Wiley-VCH.

Although the dual-cocatalyst strategy can improve OWS activity, the random deposition of HEC and OEC nanoparticles increases the likelihood of charge recombination and the water-formation reaction. The importance of separating the HEC and OEC has been suggested in some earlier studies. For instance, a Ta_3_N_5_ hollow-sphere photocatalyst whose inner and outer shells were loaded with Pt and IrO_2_ or CoO_*x*_, respectively, exhibited enhanced HER and OER activities compared with Ta_3_N_5_ hollow-sphere particles randomly loaded with the cocatalysts.^58^ Xu *et al.* constructed ZnTiO_3−*x*_N_*y*_ hollow nanospheres using carbon spheres as a template and selectively deposited Pt as the HEC and RhO_*x*_ as the OEC onto the inner and outer surfaces, respectively (Fig. 9).^59^ Compared with ZnTiO_3−*x*_N_*y*_ hollow nanospheres with randomly decorated cocatalyst nanoparticles, such a selective deposition method was found to improve the AQE of the one-step-excitation OWS reaction eightfold to 0.22% (420 ± 20 nm) and to increase the STH to 0.02%. The improvement in photocatalytic activity was attributed to the core–shell photocatalyst and the spatial separation of the cocatalysts, both of which can enhance the separation of photogenerated electrons and holes as well as inhibit the backward reaction. The use of hollow-sphere photocatalysts offers opportunities to separate the cocatalyst loading sites. However, a key issue in this approach is the difficulty associated with preparing single-crystal shells. If the photocatalyst shell is polycrystalline, it will contain grain boundaries; thus, charge separation might not be promoted even if cocatalysts are loaded with good spatial separation.

## Conclusion and outlook

**Fig. 10:**
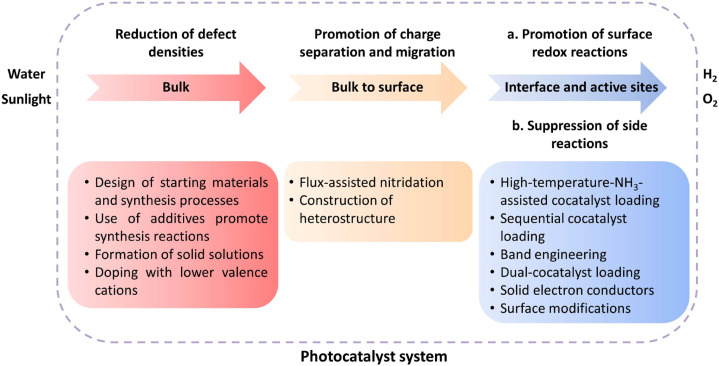
Summary of challenges and recent strategies.

Over the past decade, numerous transition-metal (oxy)nitride semiconductors capable of driving the OWS reaction *via* the Z-scheme or one-step excitation have been reported. Traditionally, efforts have been devoted to reducing the defect density while suppressing the excessive growth of particles in the development of transition-metal (oxy)nitrides because they often contain defects as a result of harsh nitridation conditions (Fig. 10). To this end, modifications of precursors and refinements of the preparation conditions (*e.g.*, flux-assisted nitridation and aliovalent doping) and post-treatment (*e.g.*, surface modifications) have been applied, partially solving the problem. Additional efforts have been devoted to improving charge separation/migration and surface redox reactions while suppressing side reactions through surface modifications. However, the STH achieved with existing (oxy)nitride photocatalysts falls far short of the goal (>5%). Given the AQY values (<7% for Z-scheme OWS and ≤0.02% for one-step-excitation OWS), only a very small portion of photons are successfully used to split water. Thus, establishing strategies that can enhance charge separation/injection and suppress side reactions is critical.

Much room remains for further improving the synthesis of transition-metal (oxy)nitrides (Fig. 10). Such improvements will remain an important approach to enhancing the OWS activity to reduce the particle size, because smaller particles shorten the charge carrier migration distance and, in principle, lower the probability of recombination in the bulk of the material if the crystallinity and the defect density are not deteriorated. One approach to balancing small particle size and high crystallinity is to synthesize one-dimensional (rod-like) or two-dimensional (sheet-like) transition-metal (oxy)nitride single-crystal nanoparticles and exploit their anisotropic crystal structures. For example, single-crystalline Ta_3_N_5_ nanorods generated directly on the edges of KTaO_3_ particles have been reported to split water without any sacrificial agent, whereas bulk Ta_3_N_5_ was inactive.^60^ In addition, given the profound effect of starting materials on the particle and material properties of transition-metal (oxy)nitrides obtained by thermal nitridation, a survey of starting materials and detailed examinations of the nitridation process might provide clues for dramatically improving the synthesis of photocatalyst materials.

Another promising strategy is the site-selective loading of cocatalysts, which has been applied to oxide photocatalysts, resulting in a substantial improvement in their OWS activity. However, this strategy has not been successfully applied to transition-metal (oxy)nitrides. A major challenge is how to prepare (oxy)nitrides with high crystallinity and exposed anisotropic facets.

Regarding the Z-scheme OWS system, when (oxy)nitrides are used as either the HEP or OEP, oxide photocatalysts are used as their counterpart in most cases. Utilizing a wider range of visible light necessitates the development of a Z-scheme OWS system solely involving (oxy)nitride photocatalysts with an absorption-edge wavelength greater than 600 nm. To this end, the design of (oxy)nitrides that can drive the HER and/or OER efficiently and with high selectivity (*i.e.*, without promoting reverse or side reactions) is also desirable. The photocatalyst sheet system based on HEPs and OEPs fixed with conductive materials is a particularly promising approach to realizing efficient Z-scheme OWS, given the relatively high STH and potential scalability demonstrated for some oxide photocatalysts. An STH beyond 1% has been achieved by a SrTiO_3_:La,Rh/C/BiVO_4_:Mo photocatalyst sheet even under ambient pressure.^61^ However, the highest STH achieved with photocatalyst sheets based on transition-metal (oxy)nitrides is 3.5 × 10^−3^% for (RhCrO_*x*_/ZrO_2_/LaMg_1/3_Ta_2/3_O_2_N)/(Au,RGO)/BiVO_4_:Mo.^44^ A better understanding of the low activity is needed, and the photocatalytic activity should be further improved through appropriate design. Research into solid-state electron conductors is important but is still in progress and immature.^15,62^ A photovoltaic-electrochemical (PV-EC) solar hydrogen production system is also a choice for transition-metal (oxy)nitride semiconductors because significant progress has been achieved in such systems.^63^ However, approaches to scale up the system and reduce manufacturing costs should also be identified.

Overall, transition-metal (oxy)nitrides constitute a promising group of materials for OWS catalysts because their absorption-edge wavelength is sufficiently long to meet the STH target and their band positions can be controlled. We hope this perspective will facilitate progress in this field and bridge the gap between what researchers have achieved and what society needs for practical solar hydrogen production *via* photocatalytic water splitting.

## Author contributions

Kaihong Chen: conceptualization, writing original draft, writing review & editing; Jiadong Xiao: conceptualization, writing review & editing; Takashi Hisatomi: conceptualization, writing review & editing; Kazunari Domen: conceptualization, funding acquisition, writing review & editing.

## Conflicts of interest

The authors declare no competing financial interest.

## Supplementary Material
